# Model-based analysis of thinking in problem posing as sentence integration focused on violation of the constraints

**DOI:** 10.1186/s41039-017-0057-5

**Published:** 2017-06-07

**Authors:** Ahmad Afif Supianto, Yusuke Hayashi, Tsukasa Hirashima

**Affiliations:** 1 Department of Information Engineering, Graduate School of Engineering, Hiroshima University, 1-4-1 Kagamiyama, Higashi-hiroshima, Hiroshima 739-8527 Japan; 2 Department of Informatics, Faculty of Computer Science (FILKOM), Brawijaya University, 8 Veteran Road, Malang, 65145 Indonesia

**Keywords:** Problem-posing process, Intermediate products, Arithmetic word problems, Learning analytics

## Abstract

The advancement of computer and communication technologies has enabled researchers to conduct and analyze the learning process of posing problems. This study investigates what learners think while posing problems as sentence integration in terms of intermediate products as well as the posed problems as the resultant product. Problem posing as sentence integration defines the arithmetic word problem structure, and posing a problem is a task to satisfy all the constraints and requirements to build a valid structure. A previous study shows that, in problem posing as sentence integration for arithmetic word problems, learners try to satisfy a relatively large number of constraints in the posed problems. In contrast, this study focuses on the violation of constraints in the intermediate products while posing problems. The result shows that learners were inclined to avoid as many violated constraints as possible throughout the problem-posing process. Although learners tend to avoid the violated constraints, naturally, they cannot avoid some mistakes. Further analysis shows that learners actually have difficulty in fulfilling particular constraints while posing the problems. Based on this analysis, it is possible to detect the difficulty of learners’ actions from the model perspective. Hence, it is possible to give accurate feedback and appropriately support the learners.

## Introduction

Problem posing is recognized as a key component in the nature of mathematical thinking (Kilpatrick 1987). Posing a problem involves generating new problems and questions aimed at exploring a given situation as well as reformulating a problem during the course of solving a related problem (Silver 1994). The development of problem-posing skills for learners is one of the main aims of learning mathematics, and it should occupy a significant role in mathematical activities (Crespo 2003). There is an increased emphasis on providing learners with opportunities for posing problems in the mathematics classroom (Stoyanova 2005; Singer et al. 2011; Cankoy 2014). Several investigations have confirmed that learning by problem posing in classrooms is a promising activity in learning mathematics (Silver and Cai 1996; English 1998). The quality of problems that learners generate depends on the given assignments (Leung and Silver 1997). In posing a problem, assessment of each problem and assistance based on it are necessary (Hirashima et al. 2007). Teacher assessment of posed problems encompasses learners’ development of diverse mathematical thinking processes (English 1997). Since learners are usually allowed to pose several kinds of problems in a broad range, it can be challenging for teachers to complete the assessment and feedback for the posed problems in classrooms.

To address this issue, technology-enhanced approaches have been conducted to realize learning by problem posing in a practical way, especially regarding assessment and feedback. Self- and peer-assessed posed problems were examined to determine the effect of learners’ self-assessment of their mathematical creativity (Shriki and Lavy 2014), to explore learner’s learning and knowledge sharing while engaged in an online question-posing and peer-assessment activity (Barak and Rafaeli 2004) and to determine which peer-assessment mode(s) students perceive most positively using student generation of questions (Yu 2011). In contrast, diagnosis functions that can automatically assess and provide feedback for each posed problem have been proposed (Nakano et al. 1999; Hirashima et al. 2000). This automatic method of diagnosis-facility assessment is called agent assessment. Furthermore, a learning system named Monsakun, which uses agent assessment for operations of addition and subtraction, has been developed (Hirashima et al. 2007). The system has many problem-posing assignments and requests learners pose the required problem by combining three simple sentences from given sentences until they successfully pose the required problem in each assignment. Using this system, the opportunity to pose the problems for learners increases. The feedback to learners according to their mistakes is provided, and for teachers, checking the validity of the posed problems becomes easier. This study aims at analyzing the practical realization of agent assessment to understand the learning process of posing problems.

Using Monsakun as a problem-posing learning system, learners’ abilities to solve problems as well as to understand them are promoted. In practical use and long-term evaluation, it was confirmed that learning by problem posing with Monsakun is interesting and useful as a learning method (Hirashima et al. 2008). Lectures and exercises with Monsakun improve not only learners’ problem-posing skills but also their problem-solving skills (Yamamoto et al. 2012). Through previous research, the usefulness of Monsakun has been confirmed for learning through posing problems. The basis of Monsakun is the triplet structure model (Hirashima et al. 2014) that defines the structure of an arithmetic word problem using sentence integration. This model deals with an arithmetic word problem that is solved using only one arithmetical operation. This is the fundamental unit of conceptual quantity representation, and much more complex arithmetic word problems can be composed by the combination of the units. An arithmetic word problem in this model is an integration of three sentences representing numerical concepts. In addition to that, the model defines constraints for valid problems that must be satisfied. When a learner can pose the required problem in Monsakun, the problem certainly meets the constraints. In other words, posing problems in Monsakun is the division of the task to pose an arithmetic word problem into two sub-tasks: generation and integration of three sentences satisfying the required constraints and the replacement of the generation (sub-) tasks by selecting tasks of sentences. This is the same as the concept of the “kit-build concept map” and focuses learner’s thinking processes on the structure of the learning content (Hirashima et al. 2015).

Although the usefulness of Monsakun has been confirmed for learning by problem posing, it is necessary to investigate the validity of the learners’ problem-posing process in Monsakun. There are two main points that explain the necessity of the investigation in this study. First, the previous study reported that although learners gave many wrong answers to get the correct answer in some assignments, they did not pose the required problems randomly, and their many wrong answers are not meaningless as the results of thinking (Hasanah et al. 2015b). In the study, problem posing as sentence integration is presumed from the trends of posed problems as the result of the process. However, we extend the analysis by involving the process of arranging the problem. We assume that learners must think the constraints form a valid problem throughout the problem-posing process. In regard to the process in posing problems, further analysis demonstrated that learners attempted to pose problems to satisfy as many constraints as possible based on their own understanding (Supianto et al. 2016a). In contrast, this study investigates the problem-posing process and reveals the trends of the process, focusing on the violation of constraints. We conduct this study to prove that learners tend to avoid as many violated constraints as possible in composing problems.

Second, the fact that learners gave wrong answers illustrates that learners cannot avoid some mistakes. Therefore, it is essential to understand the learners’ difficulties while posing the problems. Supianto et al. (2016b) detected important circumstances in the situation in which learners experience bottlenecks and misunderstanding of the structure of the problems. The study proposed a method to visualize learners’ actions from Monsakun log data. In contrast, this study analyzes the problem-posing process based on the Monsakun model. However, based only on the triplet structure model, it is not obvious which constraints are difficult for learners to satisfy because the model just shows all the possibilities of the principle. Therefore, this study combines the Monsakun model and Monsakun log data. This study investigates learners’ actions based on the Monsakun model. We show that learners have difficulty avoiding some specific types of constraints.

In this paper, we conduct a process analysis of elementary school students during problem-posing activities using Monsakun based on two main research questions. The research questions are (1) whether learners pose problems by attempting to avoid as many violated constraints as possible and (2) whether learners have difficulty in avoiding a particular type of constraints.

### Related work

In recent years, interest in integrating problem posing in mathematical instruction has continuously grown among mathematics education researchers and practitioners (Norman 2011; Ellerton 2013; Singer et al. 2015; Cai and Jiang 2016). Investigations of problems posed by learners and teachers in classrooms have provided insight into the relationships between mathematical knowledge, skills, and processes (Chen et al. 2011; Stickles 2011; Kılıç 2013; Van Harpen and Presmeg 2013). Given the importance of problem-posing activities in school mathematics, some researchers have investigated various aspects of problem-posing processes. One important direction is to examine thinking processes related to problem posing (e.g., Bonotto 2013; Şengül and Katranci 2015). Other studies underline the need to incorporate problem-posing activities into mathematics classrooms to determine prospective teachers’ problem-posing skills appropriate to selecting, translating, comprehending, and editing models and the possible difficulties they could encounter during the process in fraction problems (Işık et al. 2011), to explore students’ creativity in mathematics by analyzing their problem-posing abilities in geometric scenarios (Van Harpen and Sriraman 2013) and to examine the knowledge influences of learners’ abilities in posing combinatorial problems (Melušová and Šunderlík 2014). Furthermore, some studies provide evidence that problem posing has a positive influence on students’ abilities in problem solving (e.g., Kar et al. 2010; Şengül and Katranci 2012). Kar et al. (2010) asserted that the positive relation between posing and solving problems is an indicator of the acceptance of problem-posing skills as a phase in the development of problem-solving skills. In the analysis of the posed problems, the participants map the level of their own notions and concepts, understanding, and various interpretations and realize possible misconceptions and erroneous reasoning (Tichá and Hošpesová 2009). Learning to pose problems might also enhance learning to understand mathematical concepts (Pirie 2002). Pirie (2002) said that in asking questions on mathematical concepts, students might come to understand those concepts in a more generalized, less context-dependent way. In addition, Toluk-Uçar (2009) emphasized that problem posing has a positive effect on understanding fractions as well as on learners’ views about what it means to know mathematics.

On the other hand, investigations of problem posing from the viewpoint of interactive learning systems promote active engagement in learning through the activities of learners. Chang et al. (2012) developed game-based problem-solving modules in a mathematics problem-posing system and investigated the effects of the problem-posing system on students’ abilities to pose and solve problems. Yamamoto et al. (2012) and Abramovich and Cho (2015) demonstrated how the appropriate use of digital technology tools can motivate problem-posing activities and evaluate the learner’s performance by assessing the number of posed problems. Hung et al. (2014a) investigated the effects of an integrated mind mapping and problem-posing approach on learners’ in-field mobile learning performance in an elementary school natural science course. Moreover, Majumdar and Iyer (2015) presented how an online visual analytic tool can be used to analyze clicker responses during an active learning strategy where the instructor poses a multiple-choice question. In this study, an interactive learning system is used to encourage learners in posing arithmetic word problems. The system asks learners to arrange and integrate five or six presented sentence cards into a problem, which consists of three sentence cards. We analyze the learners’ tendencies while posing the problems in the system.

Several studies examined learners’ behaviors through a collaborative problem-posing strategy. Beal and Cohen (2012) demonstrated that the mathematics problem-posing skill was improved when the activity was carried out over an online collaborative learning system. Mishra and Iyer (2015) implemented a collaborative problem-posing activity in which two learners collaborated as a team to generate questions. Sung et al. (2016) conducted a group collaborative problem-posing mobile learning activity. They found that such an approach could improve learning achievement and group learning self-efficacy. In this study, we analyze log data of learners’ individual activity collected from a tablet personal computer-based software for learning by posing arithmetic word problems.

Several problem-posing techniques on interactive learning systems have been conducted. One approach is using the question-posing technique. The systems allow students to generate different types of questions using different media formats with peer-assessment using one type of communication mode (Wilson 2004) and multiple peer-assessment modes (Yu 2011). The studies evaluated students’ abilities to pose questions and their processes in an online learning system. Lan and Lin (2011) developed a system integrating a reward mechanism into assessment activities and analyzed student’s abilities to pose questions in a web-based learning system. Moreover, Hung et al. (2014b) investigated the effect of promoting questioning ability in problem-based scientific inquiry activities. The research developed a ubiquitous problem-based learning system regarding learners’ question-raising performance. This study used agent assessment, which can assess the validity of posed problems and automatically give feedback to the learners according to their mistakes. We investigate the learners’ difficulty based on their actions, which are logged in the system.

The second approach is learning from the example technique. This support system is developed to facilitate posing of diverse problems by learners using examples. Leikin (2015) described posing various types of problems associated with geometry investigations using examples from a course with prospective mathematics teachers, while Hsiao et al. (2013) conducted examples across three homework exercises in which students were required to generate at least one applied problem. The studies showed that integrating worked examples into problem posing has a significant skill development effect on posing more oriented and complex problems. Moreover, Kojima et al. (2015) presented examples that are merely shown to the learners and prompted them to compare the base with their posed problems. They investigated the effects of learning from an example on solution composition for posing problems. The system used in this study provides sentence cards and requests learners to create a problem according to the requirements in the task. The learners’ activities while arranging the sentence cards are recorded by the system. Then, we check their thinking processes in posing the problem focused on violation of the constraints.

Another approach is learning by problem posing as sentence integration. Problem posing as sentence integration requires learners to interpret the sentence cards and integrate them into one problem. In an assignment, the system presents a requirement, which consists of a story type and a numerical expression. The system asks learners to arrange the provided sentence cards based on the requirement. One of the few research studies that has been found in this direction is about analyzing the results of the posed problems. Hirashima et al. (2007) examined whether learners could pose the problems, showing and discussing the number of posed problems and correct problems based on the system log data. Kurayama and Hirashima (2010) analyzed the learning effects by comparing pre- and post-test problem-solving and problem-posing scores. Further analyses have been conducted on this topic by investigating the learners’ thinking processes based on the first selected sentence in assignments (Hasanah et al. 2015a) concerning the completed posed problems (Hasanah et al. 2015b). There is a dearth of research that investigates every action of learners in posing the problems to understand the learning process of problem posing on an interactive learning system. Moreover, no significant research has been found that examines the intermediate products while posing the problems. In this study, we examine every learner’s movements while posing an arithmetic word problem.

There has been considerable thorough and fine-grained investigation of the activities of learners in interactive learning systems to reveal their behavior throughout the learning process. Fournier-Viger et al. (2010) developed a virtual learning system for learning how to operate the Canadarm2 robotic arm on the international space station. The study extracted patterns from learners’ solutions to problem-solving exercises for automatically learning a task model that can then be used to aid and guide them during problem-solving activities. Hou (2012) utilized an online discussion activity adopting a role-playing strategy and conducted an empirical analysis to explore and evaluate both the content structure and behavioral patterns in the discussion process. The study adopted a new method of multi-dimensional process analysis that integrates both content and sequential analyses, whereby the dimension of interaction and cognition are analyzed simultaneously. Hsieh et al. (2015) identified higher and lower engagement patterns to represent students’ learning processes in a game-based learning system. The study investigated a possible connection between students’ verbal (asking themselves, expressing frustration, etc.) and nonverbal (smiling, focusing, moving closer to the screen, moving away from the screen, etc.) behaviors. However, the central issue in such research is basically limited to solving problems and does not include posing problems.

This study aims to investigate the problem-posing process and reveals the trends of the process. Problem-posing activities could provide us with valuable insight into a learner’s understanding of mathematical concepts and processes. Studies in this area suggest that problem posing has a positive influence on a learner’s ability to solve problems. There is significant improvement in the problem-solving performance of learners. In addition, problem posing could guide learners to achieve understanding of mathematical concepts. Technology-enhanced learning has been developed to realize and actively promote learning by problem posing. Several methods of problem-posing activities on an interactive learning system have been proposed, such as posing questions, learning from examples, and learning by problem posing as sentence integration. Additionally, considerable studies have been analyzing the results of posed problems and the learning effects. Moreover, investigational studies that examine the process of a learner’s activities in an interactive learning system to reveal behavior have been conducted, and deep examination of learner behaviors may make beneficial contributions to the educational technology field with the adoption of process analysis. This study investigates the problem-posing processes of Japanese elementary students in actual classes by analyzing the log files of the learners’ problem-posing activities on a computer-based learning system with sentence integration, which is called Monsakun.

## Methods

### Participants and procedure

In this research, we analyze the Monsakun log data of 39 first-grade students who participated in the practical use of Monsakun; their average age was 6 years old. In practical use, as described by Yamamoto et al. (2012), Monsakun was introduced as a problem-posing system of arithmetic word problems at the beginning of class (5–10 min). The teacher distributed tablets containing Monsakun to learners and explained how to operate the system. Then, the teacher taught problem structures by simulating an assignment on the blackboard (20–35 min). The teacher provided several sentence cards from Monsakun problems and conducted a lesson that resembled the Monsakun problem-posing process. The teacher encouraged participation and active discussion from all learners to pose the correct answer together. Finally, at the end of class, learners used Monsakun to complete an exercise in posing the problems individually (5–10 min). We collected the log data from the activity at this time.

Monsakun has five levels of problems that require different thinking approaches. All levels are the same in terms of posing problems from a card set, but they have different requirements. Levels 1–4 provide the numerical formula of the story, while level 5 is required to consider the unknown number. There are 12 assignments and four story types in the level: combination stories (assignments 1–3), increase stories (assignments 4–6), decrease stories (assignments 7–9), and comparison stories (assignments 10–12). An assignment is completed when learners pose the problem correctly. As a feature of Monsakun, each time a learner makes a mistake, the system will provide explanation feedback according to the mistake. This feedback will stimulate the learner to think about the other solutions and lead them to the correct answer.

### Problem-posing activity in Monsakun

**Fig. 1:**
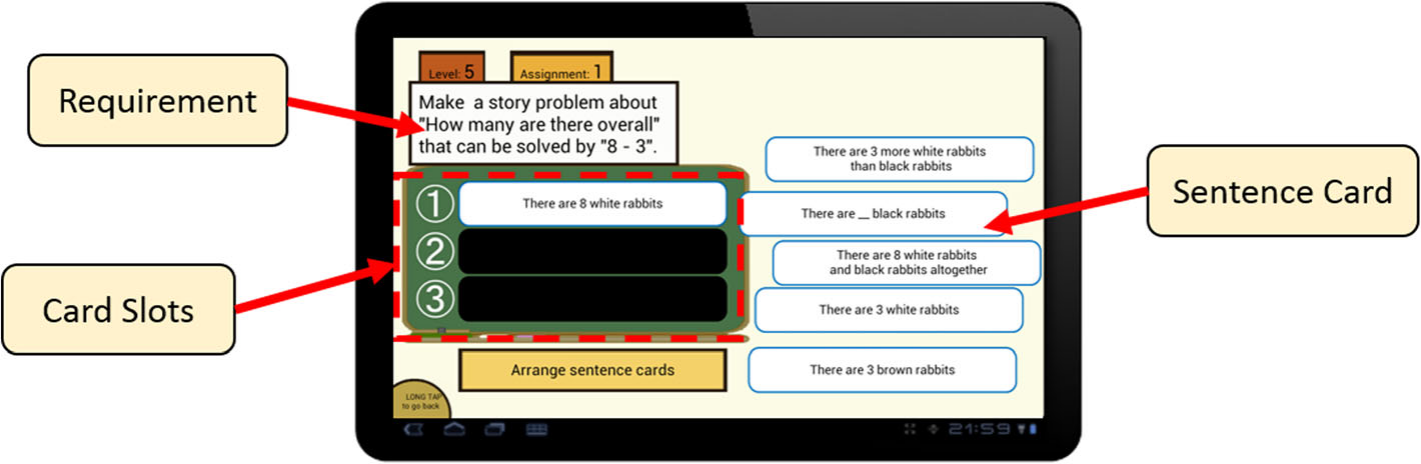
Interface of Monsakun

The interface of Monsakun is shown in Fig. 1. In the problem-posing activity using Monsakun, the learners do not create their own problem statements; however, they are required to interpret the sentence cards and integrate them into one problem in the card slot part. This activity is called “problem-posing as sentence-integration” (Hirashima et al. 2007). The system provides a set of sentence cards and a numerical expression in the requirement part, and then learners pose an arithmetic word problem based on the triplet structure model using the numerical expression by selecting and arranging appropriate sentence cards.

The triplet structure model defines an arithmetic word problem solved by addition or subtraction as a composition of three simple sentences with two “existence sentences” and one “relational sentence.” An existence sentence represents a number of single objects that has an independent quantity. A relational sentence has a relative quantity and contains a keyword that represents a story type. Although an existence sentence can be used in any story, each type of relational sentence is used only in one type of story. There are four story types: combination story, increase story, decrease story, and comparison story.

**Fig. 2:**
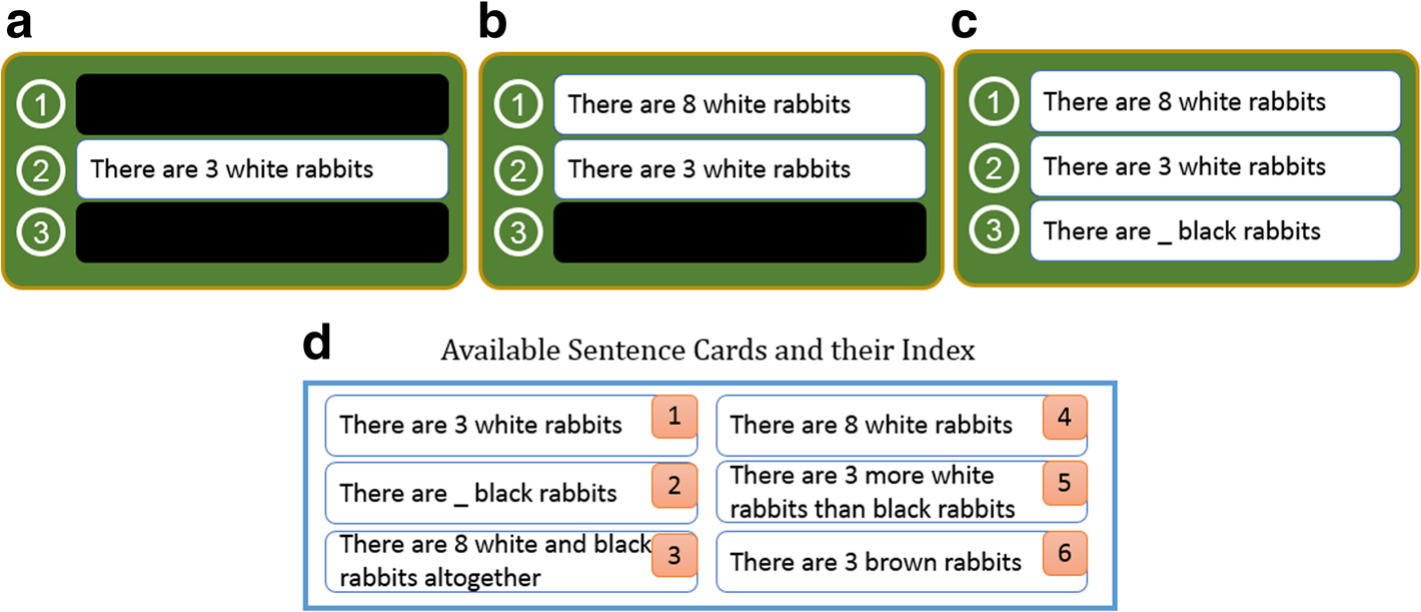
Example of states and the index of available sentence cards. **a**–**c** States. **d** Available sentence cards and their indexes

Monsakun records learners’ problem-posing activity as a combination of sentence cards in the card slots. The product of a problem-posing activity is the result of selecting and arranging a sentence card in the card slot or removing a sentence card from the card slot, which is called a “state.” When the product is composed of three sentence cards (the card slots are completely arranged), then it is called the “posed problem.” An example of the posed-problem condition is shown in Fig. 2c, whereas when the product is not composed of three sentence cards, then it is called the “intermediate product,” which is in the process of posing the problem. The examples of the intermediate products are shown in Fig. 2a, b.

The sentence cards are encoded with an indexing number shown in Fig. 2d. When the slot is still empty, index = 0 is implemented. For instance, when learners pose the problem by selecting sentence card #1 and arrange it into the second slot, state 010 has been obtained, which is shown in Fig. 2a. Another example of a state is shown in Fig. 2b; state 410 happens when learners pose the problem by selecting sentence Card #4 and then arranging it into the first slot and selecting sentence card #1 and then arranging it into the second slot.

**Fig. 3:**
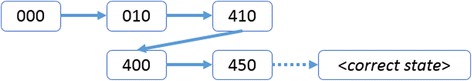
Several states generated from a learner’s steps

To complete an assignment, the learners attempt to arrange various combinations of sentence cards to generate a particular state according to what they set. They arrange the composition until they reach the composition of the correct answer. For instance, several steps performed by a learner are shown in Fig. 3. First, state 000 is generated as the initial state. In the first step, the learner begins with state 010; this means that the learner has selected the first sentence card and arranged it into the second slot. In the second step, state 410 was composed, which means the learner selected the fourth sentence card and arranged it into the first slot. In the next step, the learner removed the first sentence card from the second slot; this condition changes the state to 400. Then, the learner tries to pose the problem resulting in state 450, and so on, until the correct state is reached.

**Fig. 4:**
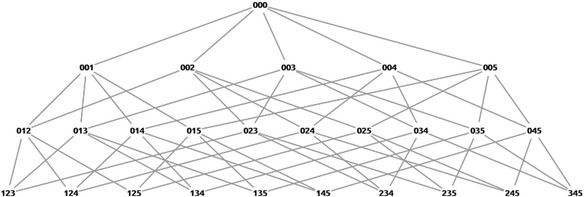
Problem state spaces of a combination story problem with five available sentence cards

According to the model, all possible combinations of sentence cards and transitions among them can be clearly defined as a network of states. We call this network “problem states space.” All the steps of posing problems in Monsakun could be mapped into a transition from one state to another in this network. All possible states that consist of three sentence card indexes are obtained by combining all available sentence cards, including index = 0. Each state represents a basic unit of thinking, and a problem state space provides the range of thinking in a problem-posing assignment. Combination and comparison stories are story problems in which the order of the sentence cards in the slot is not necessary, while increase and decrease story problems restrict the order of the cards (Supianto et al. 2016b). An example of all possible states from five available cards in a combination story is shown in Fig. 4.

### Assessment of products: constraints to form a problem

The task model of posing problems via sentence integration has been developed based on the consideration of problem types in the triplet structure model (Kurayama and Hirashima 2010). Based on the task model, five main constraints must be satisfied by each posed problem; they are (1) calculation, (2) story type, (3) number, (4) objects, and (5) sentence structure. The calculation is the numerical expression representing the story type. Calculation structure requires numbers assigned to the correct sentence structure, whether an existence sentence or relational sentence according to the story type. The story type is one of the four available story types. They are combination story, increase story, decrease story, and comparison story. The story type should be identified in the requirement. The number is the quantity in the sentence. Number structure requires the consistency of numbers in the problem. Each number in the problem must be derived from the other numbers. The object is the entity in the sentence. Object structure also requires the consistency of entities in the sentences. For example, if the story type is increase or decrease, the objects in the three sentences must be the same. On the other hand, if the story type is combination or comparison, objects in the independent quantity sentences are different, and both are in the relative quantity sentence. The sentence structure is the composition of sentences. As defined in the triplet structure model, an arithmetic word problem must consist of two existence sentences and one relational sentence. The type of relational sentence is related to the story types. When less than five constraints are satisfied, the posed problem is not valid; that is, the problem cannot be solved, or it is not the required one. The validity is measured based on the number of satisfied constraints.

The example of several states and their satisfaction of constraints in assignment 1 is presented in Table 1. The requirement of assignment 1 is *make a story problem about “How many are there overall” that can be solved by “8 − 3,”* which is an arithmetic word problem with a combination story type. There are six available sentence cards that could be used by the learners. The sentences for each card are composed of the following:

1. There are 3 white rabbits;
2. There are ? black rabbits;
3. There are 8 white and black rabbits altogether;
4. There are 8 white rabbits;
5. There are 3 more white rabbits than black rabbits; and
6. There are 3 brown rabbits.

**Table 1:** Example of several states and their satisfaction of constraints

No.	State	Composition of sentence cards	Constraint					Number of violated constraints
			C1	C2	C3	C4	C5	
1	001	–						
		–	0	0	0	0	0	0
		There are 3 white rabbits						
2	014	–						
		There are 3 white rabbits	−1	0	0	0	0	1
		There are 8 white rabbits						
3	246	There are ? black rabbits						
		There are 8 white rabbits	−1	−1	1	−1	−1	4
		There are 3 brown rabbits						

*C1* calculation, *C2* story type, *C3* number, *C4* object, *C5* sentence structure

The posed problem represented as state 246 (see Table 1, No. 3) has the validity equal to 1 because the state only satisfies one constraint (number). The state consists of numbers that fit the requirement; they are 8, 3, and the unknown number (?). However, the calculation cannot be made because it is necessary to transform the numerical expression, “8 − 3,” into the numerical expression representing a combination story, “3 + ? = 8.” In that formula, the number “3” and the unknown number “?” should be assigned as existence sentence cards, and the number “8” should be assigned as a relational sentence card, but the number “8” is an existence card on that state. Regarding the story-type constraint, there is no relational sentence card that indicates a combination story type. Then, the object also does not satisfy the constraints because all three objects are different, and they are not connected to each other. Finally, to satisfy the sentence-structure constraint, the state must consist of two existence sentence cards and one relational sentence card, but the state is composed of three existence sentence cards.

According to the triplet structure model, we only can measure the validity of the posed problem products, which is based on the number of satisfied constraints. Therefore, to cover the measurement of the intermediate products, we define three values for each constraint: −1, 0, and 1. The value of −1 indicates the constraint is violated, and the value of 0 indicates the constraint is not violated, while the value of 1 indicates the constraint is satisfied. The number of violated constraints is obtained by counting how many constraints are violated.

**Fig. 5:**
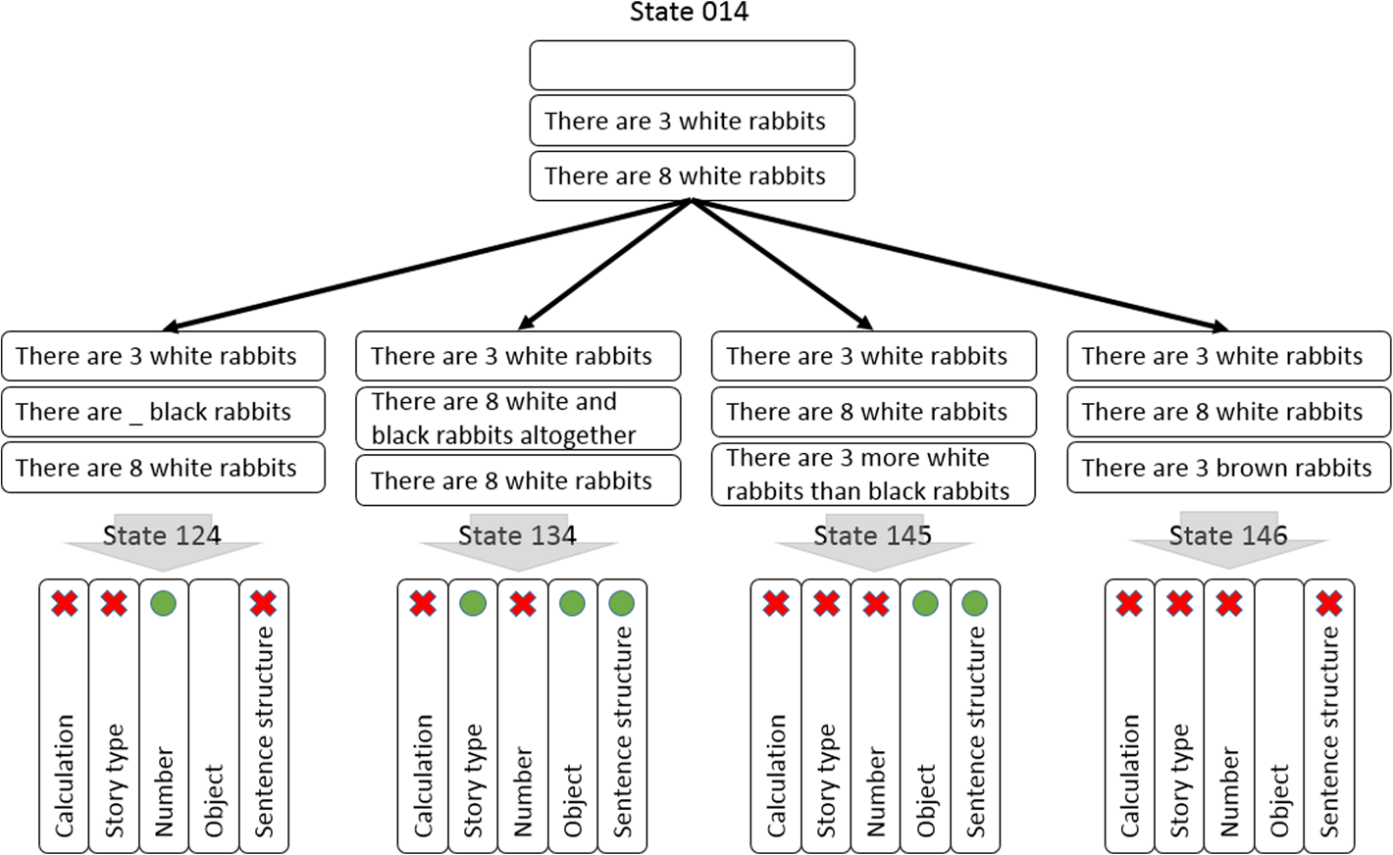
Derivative states of state 014 and the constraint satisfaction in assignment 1

Regarding the violated constraint, three states shown in Table 1 are explained. There is no satisfied constraint, nor violated constraint at the first example, state 001. This condition allows the calculation constraint to not be violated. The story type, number, object, and sentence structure are also not violated. Therefore, all constraints in this state are assigned to 0. The second example is state 014, which violates the calculation constraint. Based on the numerical expression in the requirement, the number 8 should be on the relational sentence. However, sentence card #4 is an existence sentence card, containing the number 8. Therefore, this state violates the calculation constraint, and this constraint is assigned to −1. The story type, number, object, and sentence structure are not violated nor satisfied because we still cannot determine them. Thus, the four constraints are assigned to 0. There are four states that can be derived from state 014, and each will not meet the correct state. The problems facing the derivative states are illustrated in Fig. 5. Like state 014, all derivative states will at least violate the calculation constraint. The first derivative state is state 124. Besides the calculation constraint, this state violates the story-type constraint due to the lack of story and sentence structure because all arranged sentence cards are existence cards. The second and third derivative states are states 134 and 145. Both violate calculation and number constraints. The difference between the states lies in the relational sentence card. state 134 contains a relational card that fits the required story type (combination story), while State 145 forms the comparison story type. Lastly, none of the constraints are satisfied in the derivative state 146. Four constraints are violated and one constraint is not violated or satisfied.

The last example in Table 1, state 246, satisfies only one constraint, the number constraint. There is no story that can be built from this composition, nor can the calculation and sentence structure be built. It can be calculated and well-structured when it consists of two existence sentences and one relational sentence, instead of all sentence cards being existence cards. In addition, there is no relation between objects in the composition of the sentence cards. They are independent objects consisting of white, black, and brown rabbits. This condition causes the number of violated constraints to be four because there are four constraints that are violated.

**Fig. 6:**
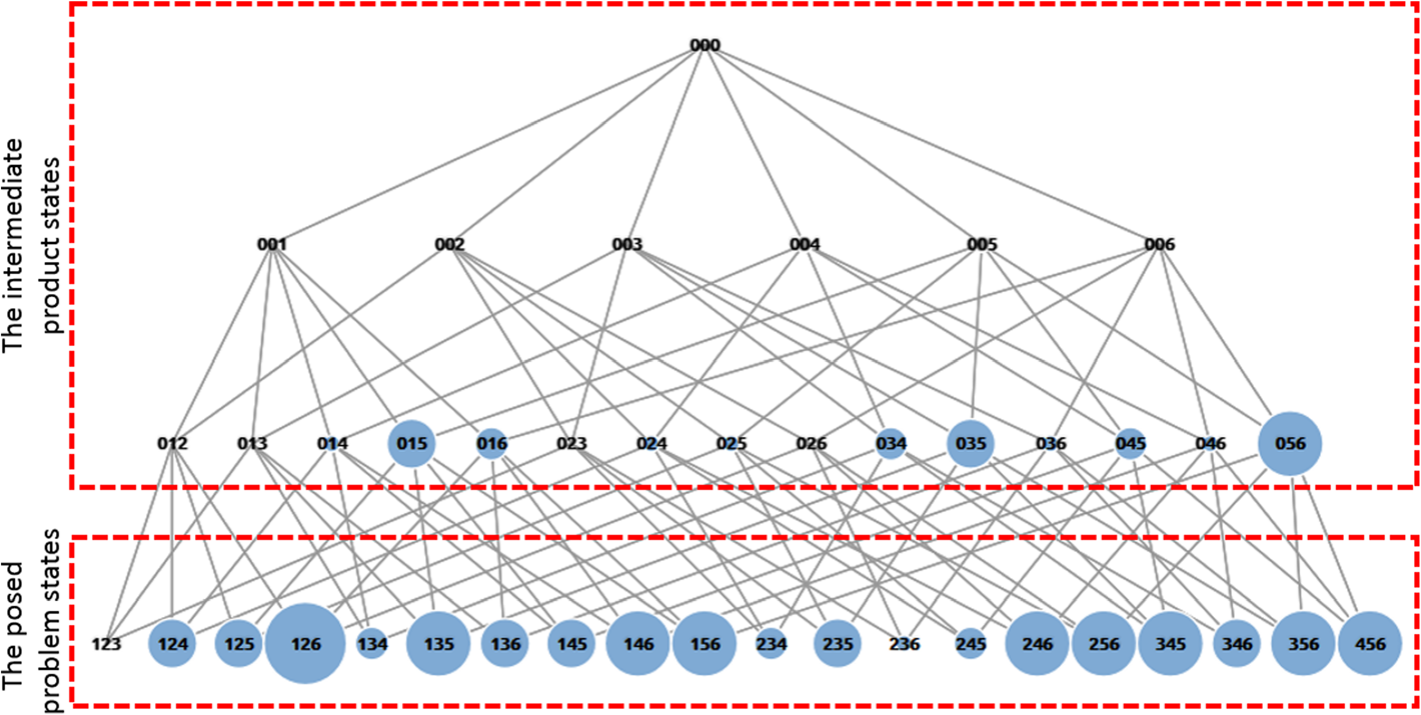
Graph of the number of violated constraints of products in assignment 1

We calculate the number of violated constraint values for all states and present the visualization that is shown in Fig. 6. The graph in Fig. 6 allows us to visualize the number of violated constraints of states presented by the size of the nodes. The larger nodes have a higher number of violated constraints than the smaller nodes. Of course, states including only the required sentence cards, #1, #2, and #3, have no violation. On the other hand, even if a state includes dummy cards, #4, #5, and #6, it can have no violation, for example, state 026. Finally, although the state cannot satisfy all the constraints in this assignment, the state is judged as itself.

### Data collection

**Fig. 7:**
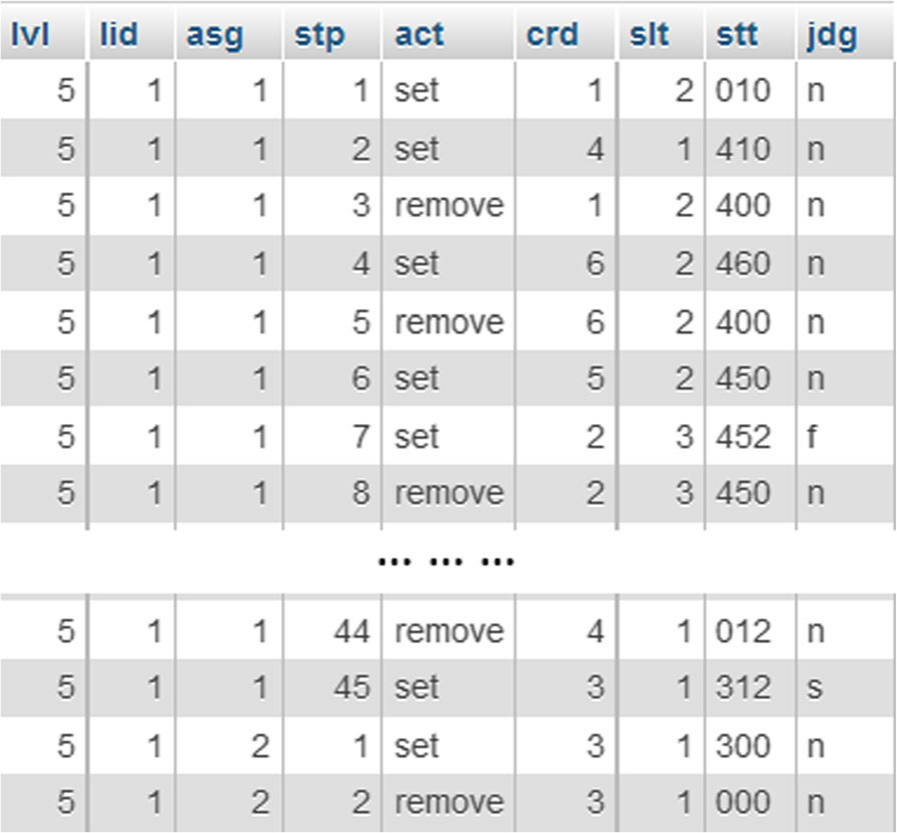
Example of log data of learners’ activity on Monsakun

The data of this study is gathered from the log files of learners’ problem-posing activities on Monsakun. Log file records the actions of learners during the learning activity using Monsakun, such as placing the sentence card, removing the sentence card, and clicking the “check answer” button. The log file consists of the learner ID and information about the activities performed in Monsakun. They are labeled lvl, lid, asg, stp, act, crd, slt, stt, and jdg. The label “lvl” is the level of assignment that determines the difficulty of the problem-posing task, and “lid” shows the learner ID. The label “asg” is the number of the assignment, and “stp” is the sequence number of the step. The label “act” consists of two possible values, set or remove, which represent placing the sentence card or removing the sentence card, respectively. The label “crd” is the index of the sentence card that is placed in the slot, which is denoted by the label “slt.” The label “stt” indicates the state generated from the combination of three sentence card indexes that is placed in the slot. The last code, “jdg,” shows the type of action, for example, incomplete state (*n*), wrong answer (*f*), or successful state (*s*). We present a sample of the log data in Fig. 7.

The analysis of learners’ performance by examining the average steps and mistakes in posing the problems on Monsakun has been reported in past research (Hasanah et al. 2015b). The average of the steps and mistakes shows how many steps a learner required to give a correct answer in one assignment and how many mistakes the learner made during the process, focusing on the posed problem states, respectively. Ideally, a learner would only need three steps to pose a correct answer because a problem in Monsakun consists of the arrangement of three simple sentence cards. The results show that the average steps and mistakes at level 5 were very high compared to the others, which shows that level 5 was indeed very challenging for learners. In this paper, investigation of every step while posing the problems was conducted, which means the intermediate product states as well as the posed problem states arranged by learners (Fig. 6) were inspected to determine the characteristics of learners at level 5 according to the violation of constraints.

### Data analysis

In Monsakun, five or six sentence cards are provided in each assignment. Three of them are correct cards, which satisfy all constraints from the assignment requirement, and when composed correctly will form the correct answer. The rest are dummy cards, which are designed through careful consideration by an expert as a meaningful distraction to the learners in order to learn the structure of a simple arithmetic word problem. Despite the nature of this learning system, it could permit learners to select three sentence cards randomly. Learners’ intention in posing the problems according to the given requirements is analyzed. Three analyses from the log files of learners’ problem-posing activity on Monsakun are conducted. We analyze their sentence card compositions. The first and second analyses provide the answer for the first research question, while the third gives the answer for the second research question.

In the first analysis, we investigate the products and conduct a bivariate correlation analysis between the occurrence frequency of the products and the number of violated constraints. The occurrence frequency shows how many states have been arranged, while the number of violated constraints shows how many constraints are violated based on the state. We assume that the degree of correlation is related to the degree of the learners’ understanding. If the number of violated constraints has a negative correlation to the frequency, then the high number of violated constraints will be followed by the lower number of actions. It means that the high number of violated compositions of sentence cards has a small number of learners’ actions. Therefore, this correlation test will provide an answer to the first research question.

The second part of the analysis investigates the portion of states in the assignment setting to the occurrence frequency. We observe differences between the number of states in the assignment setting and the occurrence frequency. Moreover, we examine the differences for each number of violated constraints. In the low-frequency violated constraints, if the portion of occurrence frequency is higher than the number of states in the assignment setting, then it expresses that learners arrange states that have low error rate. In addition, in the high-violated constraints, if the portion of occurrence frequency is lower than the number of states in the assignment setting, then it shows that learners avoid solutions that potentially have high error rate. Hence, this analysis will support providing the answer to our first research question.

Although two previous analyses show that learners tend to avoid mistakes, they still cannot avoid some mistakes, which demonstrates their difficulty in understanding the problem structure. Therefore, the third part of the analysis inspects the difficulty of learners according to the violation of constraints. We determine the ratio of the number of states in the assignment setting to the occurrence frequency. We examine the relative number of states for each type of constraint and their actual occurrence. If the number of occurrences is high, then the ratio is low. Thus, the minimum ratio in a constraint indicates that learners have difficulty avoiding such types of constraints while posing the problems. This analysis will confirm our second research question regarding whether learners have difficulty avoiding some particular type of constraints.

## Results and discussion

In this study, an investigation of learners’ actions at level 5 was conducted. We roughly analyzed every step of the learners. As mentioned in the introduction, the goal of the analysis is to address the following research questions: (RQ1) Do learners pose problems by attempting to avoid as many violated constraints as possible? (RQ2) Do learners have difficulty avoiding a particular type of constraint?

### RQ1: Do learners pose problems by attempting to avoid as many violated constraints as possible?

**Fig. 8:**
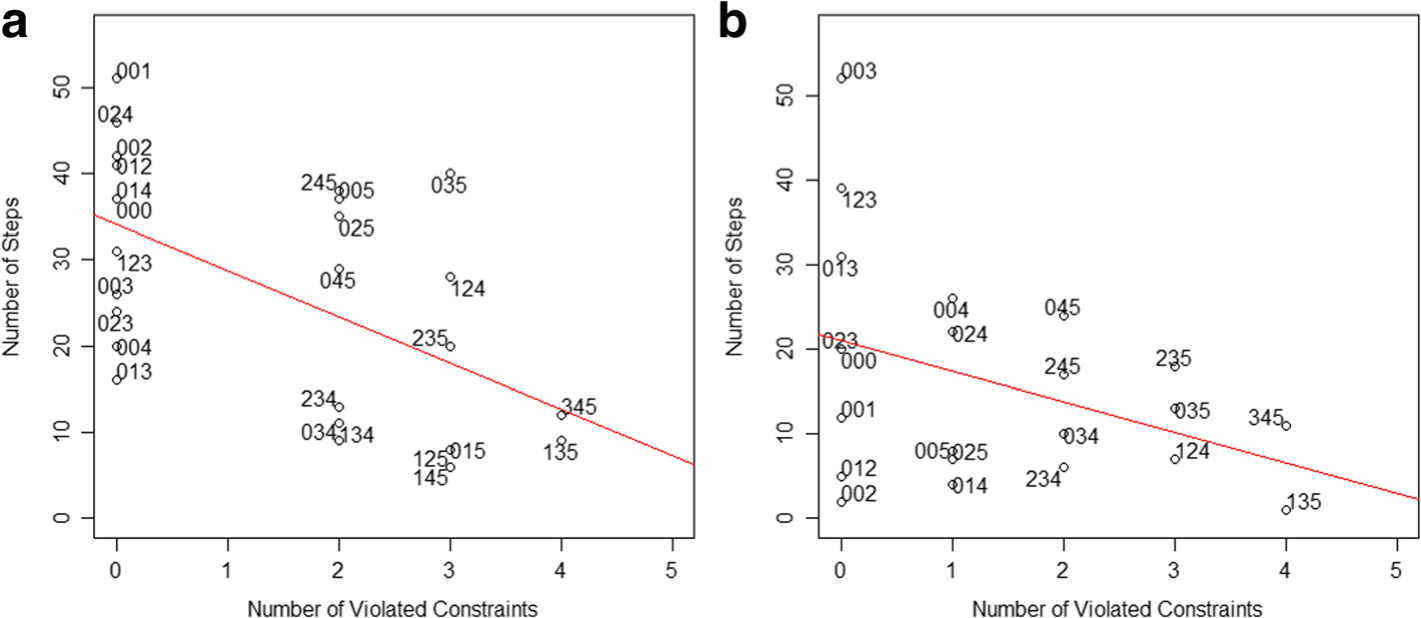
Plot of correlation between the number of violated constraints and the occurrence frequency of products. **a** Assignment 10. **b** Assignment 3

In this analysis, we conducted a Pearson’s correlation test between the number of violated constraints and the occurrence frequency of products. We evaluated both intermediate and posed problem products for each arranged state. The result is shown in Table 2. A significant correlation (*p* < 0.05) in 11 out of 12 assignments was found. Many actions performed by learners showed an inclination to avoid as many violated constraints as possible. The highest coefficient is in assignment 10 (rho = −0.5619, *p* < 0.01), and the scatterplot of this assignment is shown in Fig. 8a. The red line in the scatterplot shows the regression line of the data. Based on this information, the frequency of each product has a negative correlation with constraints violated in it. It supposed that learners attempted to arrange the problem to avoid violating more constraints. If the learners posed the problem randomly, the distribution of the number of the learners’ actions would not have a significant correlation compared to the violated constraints. This finding shows that learners were inclined to pose more valid products.

**Table 2:** Correlation analysis between the number of violated constraints and the occurrence frequency of the products

Assignment	Pearson’s correlation	*p* value
1	−0.3701*	0.0158
2	−0.4928**	0.0014
3	−0.3879^+^	0.0745
4	−0.2565**	0.0033
5	−0.2778**	0.0051
6	−0.3460**	4.51E−05
7	−0.4006**	1.35E−06
8	−0.3552**	0.0001
9	−0.3990**	4.43E−06
10	−0.5619**	0.0028
11	−0.5570**	0.0011
12	−0.4486**	0.0054

**Significant correlation (*p* < 0.01); *significant correlation (*p* < 0.05); ^+^marginal correlation (*p* < 0.1)

Furthermore, the result of correlation in assignment 3 shows marginal correlation (*p* < 0.1). The scatterplot of correlation in assignment 3 shown in Fig. 8b indicates that there is no significant difference in this assignment. Therefore, the chi-square test was conducted to determine the trends in the details. We determined the portion of the number of states in the assignment setting to the occurrence frequency based on the number of violated constraints. Here, the number of states in the assignment setting means the space of all possible compositions that can be arranged by the learners. We check the number of states that are categorized in each number of violated constraints and the occurrence frequency. We show that although the correlation between the number of violated constraints and the occurrence frequency of products is not significant, there is a significant difference between the number of states in the assignment setting and its occurrence frequency. The results of the difference analysis and the detail portion of the assignments investigated in this study are presented in Table 3. We found a significant difference in 11 out of 12 assignments (*p* < 0.01), which shows that learners made a conscious attempt to avoid more violated constraints in the assignments.

**Table 3:** Difference analysis between portions of number of states in assignment setting to its occurrence frequency

Assignment		Number of violated constraints												Setting vs occurrence	
		0	*p*	1	*p*	2	*p*	3	*p*	4	*p*	5	*p*	Chi-square	*p*
1	Setting occurrence	0.238 0.230	▼	0.190 0.370	▲ **	0.143 0.217	△ +	0.190 0.103	▼ *	0.214 0.077	▼ **	0.024 0.003	▽ +	<0.01	**
2	Setting occurrence	0.286 0.523	▲ **	0.214 0.316	▲ *	0.190 0.080	▼ **	0.214 0.071	▼ **	0.048 0.003	▼ **	0.048 0.006	▼ **	<0.01	**
3	Setting occurrence	0.308 0.510	▲ **	0.192 0.189	▽	0.192 0.161	▽	0.231 0.107	▼ **	0.077 0.034	▽			<0.01	**
4	Setting occurrence	0.324 0.541	▲ **	0.147 0.196	△	0.176 0.087	▼ *	0.265 0.167	▼ *	0.088 0.009	▼ **			<0.01	**
5	Setting occurrence	0.324 0.649	▲ **	0.191 0.191		0.265 0.044	▼ **	0.132 0.103	▽	0.088 0.013	▼ **			<0.01	**
6	Setting occurrence	0.324 0.520	▲ **	0.147 0.190	△	0.176 0.131	▽	0.265 0.151	▼ *	0.088 0.007	▼ **			<0.01	**
7	Setting occurrence	0.324 0.500	▲ **	0.147 0.189	△	0.176 0.149	▽	0.265 0.139	▼ **	0.088 0.024	▼ *			<0.01	**
8	Setting occurrence	0.434 0.696	▲ **	0.081 0.077	▽	0.265 0.117	▼ **	0.132 0.077	▽	0.088 0.034	▽ +			<0.01	**
9	Setting occurrence	0.324 0.546	▲ **	0.147 0.170	△	0.176 0.106	▽ +	0.265 0.153	▼ *	0.088 0.025	▼ *			<0.01	**
10	Setting occurrence	0.423 0.552	▲ *			0.269 0.256	▽	0.231 0.161	▽	0.077 0.031	▽ +			<0.10	+
11	Setting occurrence	0.500 0.912	▲ **	0.071 0.018	▼ *	0.143 0.055	▼ *	0.048 0.004	▼ **	0.143 0.011	▼ **	0.095 0.000	▼ **	<0.01	**
12	Setting occurrence	0.333 0.585	▲ **	0.119 0.118	▽	0.143 0.094	▽	0.262 0.180	▽ +	0.095 0.016	▼ **	0.048 0.007	▼ *	<0.01	**

**Significant difference (*p* < 0.01), *significant difference (*p* < 0.05), ^+^marginal difference (*p* < 0.1); occurrence is more than setting (▲ significant, △ not significant); occurrence is less than setting (▼ significant, ▽ not significant)

**Fig. 9:**
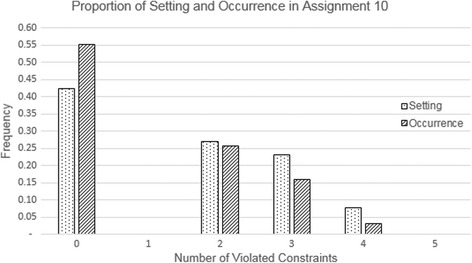
Portion of the number of states in assignment 10 to the occurrence frequency

In addition, we pay attention to the portion of the number of states in the assignment setting to its occurrence performed by the learners according to the violated constraints. We found that the occurrence frequency in the high-violated constraints is lower than the number of states in the assignment setting, while the occurrence frequency in the low-violated constraints is higher than the number of states in the assignment setting. This implies learners were trying to avoid making a composition of sentence cards with a high number of violated constraints. Moreover, we show the portion in assignment 10 (see Fig. 9), which has a marginal difference. The portion of occurrence frequency, which is more than the number of states, happens at zero violated constraints, which means that learners tried to arrange the least instances of compositions of sentence cards that could potentially have many violated constraints. This finding strengthens the previous statement that many actions of learners were aimed to avoid as many violated constraints as possible in arranging the posed problem and the intermediate products as well.

### RQ2: Do learners have difficulty avoiding a particular type of constraint?

In this analysis, we detect the difficulty of learners regarding the violation of constraints. We assume that although learners tend to avoid compositions containing violated constraints, they have difficulty avoiding a particular type of constraint. To prove it, we calculated the ratio of the number of states in the assignment setting to its occurrence frequency according to the type of constraints. The result is shown in Table 4. This ratio shows the relative sizes of the states in the assignment setting and the actual occurrence. The minimum ratio shows that learners performed many actions, which indicates they have difficulty avoiding such constraints while posing the problem. We found that, in 8 out of 12 assignments, learners have difficulty avoiding the story constraint. In addition, in 4 out of 12 assignments, they have difficulty avoiding the calculation constraint. Based on this result, we confirm that they have difficulty avoiding a particular type of constraint.

**Table 4:** Ratio of the number of states in the assignment setting to the occurrence frequency according to the type of constraints

Assignment	Type of constraints				
	Calculation	Story	Number	Object	Sentence structure
1	0.0151^a^	0.0230	0.0317	0.0469	0.0350
2	0.0971^a^	0.2308	0.3191	0.1852	0.2308
3	0.1060^a^	0.1224	0.2857		0.1190
4	0.0532	0.0458^a^	0.1341		0.0509
5	0.2145	0.2113^a^	0.6857		0.2113^a^
6	0.0633	0.0467^a^	0.1472		0.0616
7	0.0709	0.0559^a^	0.1277		0.0726
8	0.2800	0.2778^a^	0.3750		0.2778^a^
9	0.1484	0.1297^a^	0.2609		0.1449
10	0.0498	0.0448 ^a^	0.1081		0.0459
11	0.9474 ^a^	3.3333	0.9474^a^	1.5000	3.3333
12	0.1705	0.1258^a^	0.2083	0.7143	0.2500

^a^The minimum value of ratio

As previously described, level 5 is required to consider the unknown number because it is not given in the requirement. It is challenging for learners, especially in considering the story constraint. At the previous levels, there is no conflict at the required story type and numerical expression. In addition, the order of numbers in sentences is the same as the numerical expression. For instance, the requirement of level 3 assignment 1: Make a word problem about “How many are there overall” that can be solved by “4 + ? = 10,” learners can pose the required problem by arranging sentences according to the order of numbers in the numerical expression. However, this is not valid for level 5 because the numerical expression does not express the order of numbers in the required story but the solution is to evaluate the unknown number. To complete assignments at this level, for example, in the first assignment, learners need to transform the numerical expression “8 − 3” into the numerical expression representing a combination story, “3 + ? = 8.” Then, learners could assign the existence sentence cards to the number “3” and the unknown number “?.”

Investigation of learners’ activities at the process level promotes an opportunity to discover the learners’ behavior in detail. Moreover, when it is associated with a cognitive load, then the learners’ thinking processes can be explored. Particularly, what conditions learners face difficulties in attempting to pose problems could be detected. With such detections, we could define learning support depending on the learners’ mistakes and develop an adaptive function to overcome learners’ bottlenecks in attempting to pose problems.

## Conclusion

We conduct a model-based analysis of problem-posed products as well as intermediate products while posing problems from Monsakun log data of first-grade elementary school students to investigate their methods of thinking in posing arithmetic word problems. This study focuses on the violation of the constraints. The analysis involves intermediate products to prove that the learners attempt to avoid invalid intermediate products. Correlation between the numbers of violated constraints and the frequency of each intermediate product that the learners actually made was reported. Moreover, to determine the detail trends of learners’ actions, a chi-square test between the number of states in the assignment setting and the occurrence frequency was conducted. Significant correlation and difference in 11 out of 12 assignments was found, which shows that many actions performed by learners had the inclination to avoid as many violated constraints as possible. It indicates that they tended to avoid as many mistakes as possible. Furthermore, although learners tended to avoid the violated constraints, they could not avoid some mistakes. However, most of the learners’ mistakes violated at most two constraints. Further analysis shows that, in 12 assignments, learners generally have difficulty fulfilling 2 out of 5 constraints, which are “story” and “calculation” constraints. Based on this analysis, it would be possible to detect the difficulty of learners’ actions from the model perspective. Hence, accurate feedback and appropriate support can be provided.

For future research, we plan to analyze more detail about the characteristics of learners’ thinking processes. We would like to use a data-mining method, such as sequential data mining to discover learners’ action sequences while posing the problems and use the clustering method for grouping learners’ thinking processes. We also would like to explore methods to identify other significant actions. These are required to define learning support, depending on each learner’s cause of mistake and to develop an adaptive function for learning by posing problems.
